# Biochemical analysis of novel *NAA10* variants suggests distinct pathogenic mechanisms involving impaired protein N-terminal acetylation

**DOI:** 10.1007/s00439-021-02427-4

**Published:** 2022-01-17

**Authors:** Nina McTiernan, Lisbeth Tranebjærg, Anna S. Bjørheim, Jacob S. Hogue, William G. Wilson, Berkley Schmidt, Melissa M. Boerrigter, Maja L. Nybo, Marie F. Smeland, Zeynep Tümer, Thomas Arnesen

**Affiliations:** 1 Department of Biomedicine, University of Bergen, Jonas Lies vei 91, 5020 Bergen, Norway; 2 Department of Clinical Genetics, Kennedy Center, Copenhagen University Hospital, Rigshospitalet, Copenhagen, Denmark; 3 Department of Clinical Medicine, Faculty of Health and Medical Sciences, University of Copenhagen, Copenhagen, Denmark; 4 Department of Biological Sciences, University of Bergen, 5020 Bergen, Norway; 5 Department of Pediatrics, Madigan Army Medical Center, Tacoma, WA USA; 6 Division of Genetics, University of Virginia Health System, Charlottesville, VA USA; 7 Department of Medical Genetics, University Hospital of North Norway, 9038 Tromso, Norway; 8 Department of Surgery, Haukeland University Hospital, 5021 Bergen, Norway; 9 Present Address: Laboratory of Molecular Pharmacology, Department of Biomedical Sciences, Faculty of Health and Medical Sciences, University of Copenhagen, Copenhagen, Denmark; 10 Present Address: Department of Gastroenterology and Hepatology, Radboud Institute for Molecular Life Sciences, Radboud University Medical Center, Nijmegen, The Netherlands

## Abstract

NAA10 is the catalytic subunit of the N-terminal acetyltransferase complex, NatA, which is responsible for N-terminal acetylation of nearly half the human proteome. Since 2011, at least 21 different *NAA10* missense variants have been reported as pathogenic in humans. The clinical features associated with this X-linked condition vary, but commonly described features include developmental delay, intellectual disability, cardiac anomalies, brain abnormalities, facial dysmorphism and/or visual impairment. Here, we present eight individuals from five families with five different de novo or inherited *NAA10* variants. In order to determine their pathogenicity, we have performed biochemical characterisation of the four novel variants c.16G>C p.(A6P), c.235C>T p.(R79C), c.386A>C p.(Q129P) and c.469G>A p.(E157K). Additionally, we clinically describe one new case with a previously identified pathogenic variant, c.384T>G p.(F128L). Our study provides important insight into how different *NAA10* missense variants impact distinct biochemical functions of NAA10 involving the ability of NAA10 to perform N-terminal acetylation. These investigations may partially explain the phenotypic variability in affected individuals and emphasise the complexity of the cellular pathways downstream of NAA10.

**Supplementary Information:**

The online version contains supplementary material available at 10.1007/s00439-021-02427-4.

## Introduction

N-terminal acetylation (Nt-Ac) is a widespread protein modification that contributes to a greatly diversified proteome, as approximately 80–90% of human proteins are subjected to acetylation at their N-terminus (Aksnes et al. 2019; Arnesen et al. 2009). Seven N-terminal acetyltransferases (NATs) have been identified in humans, named NatA-NatF and NatH, all of which have distinct substrate specificities (Aksnes et al. 2019). Following initiator methionine cleavage, NatA co-translationally transfers an acetyl group from acetyl coenzyme A (Ac-CoA) to N-termini starting with serine, valine, cysteine, glycine, alanine, or threonine, and this group of N-termini encompasses nearly half the human proteome (Arnesen et al. 2009). The major components of NatA are the catalytic subunit NAA10 and the auxiliary subunit NAA15 (Arnesen et al. 2005; Mullen et al. 1989; Park and Szostak 1992). In addition to NAA10’s role as the catalytic subunit of NatA, a cellular population of NAA10 exists as monomers and is proposed to exert different biochemical functions (Arnesen et al. 2005; Damme et al. 2011). Monomeric NAA10 has been reported to catalyse lysine acetylation of several protein substrates, such as Hsp70, β-catenin, Runx2, MLCK and AuA (Seo et al. 2016; Lim et al. 2006; Yoon et al. 2014; Shin et al. 2009; Vo et al. 2017), some of which have been disputed (Magin et al. 2016). Moreover, NAA10 influences gene transcription through non-catalytic regulation of DNMT1 (Lee et al. 2010, 2017).

*NAA10* is located on the X-chromosome (Xq28) and is universally expressed in human tissues (Wu and Lyon 2018). Studies have shown that NAA10 dysregulation can be pathological in humans (Aksnes et al. 2019; Wu and Lyon 2018; Kim et al. 2020), and NAA10 has been associated with several human cancers both as an oncoprotein and tumour suppressor depending on the cancer type (Kim et al. 2020; Kalvik and Arnesen 2013).

In the last decade, *NAA10* missense variants have been recognised as the cause of an X-linked genetic disease known as *NAA10*-related syndrome or Ogden syndrome (OMIM #300855) (Wu and Lyon 2018). The first *NAA10* missense variant described, p.(S37P), was identified in 2011 in two unrelated families, where the hemizygous boys suffered from global developmental delay (DD) (HP:0001263), hypotonia (HP:0001252), cardiac anomalies (HP:0001627), and died within 2 years of age (Rope et al. 2011). In contrast, the heterozygous females were asymptomatic or only mildly affected (Rope et al. 2011; Myklebust et al. 2015). Subsequently, a series of additional pathogenic *NAA10* missense variants have been identified in both males and females (Casey et al. 2015; Popp et al. 2015; Saunier et al. 2016; McTiernan et al. 2018, 2021; Støve et al. 2018; Cheng et al. 2019; Ree et al. 2019; Bader et al. 2020; Gogoll et al. 2021; Maini et al. 2021). The phenotypes and disease severity differ, but the common clinical features include DD, intellectual disability (ID) (HP:0001249), and cardiac anomalies. Furthermore, many individuals also display brain abnormalities (HP:0012443), skeletal abnormalities (HP:0000924), hearing impairment (HP:0000365), visual impairment (HP:0000505) and/or facial dysmorphism (HP:0001999) (Casey et al. 2015; Popp et al. 2015; Saunier et al. 2016; McTiernan et al. 2018, 2021; Støve et al. 2018; Cheng et al. 2019; Ree et al. 2019; Bader et al. 2020; Gogoll et al. 2021; Maini et al. 2021). In this study, we present eight new individuals from five families with five inherited or de novo* NAA10* variants, four of which are novel. We have biochemically characterised the four novel *NAA10* variants, c.16G>C p.(A6P), c.235C>T p.(R79C), c.386A>C p.(Q129P) and c.469G>A p.(E157K), and clinically described one new de novo case of the previously studied c.384T>G p.(F128L) variant (Saunier et al. 2016; Cheng et al. 2019).

## Results

### Clinical report: family 1, individual 1

**Fig. 1:**
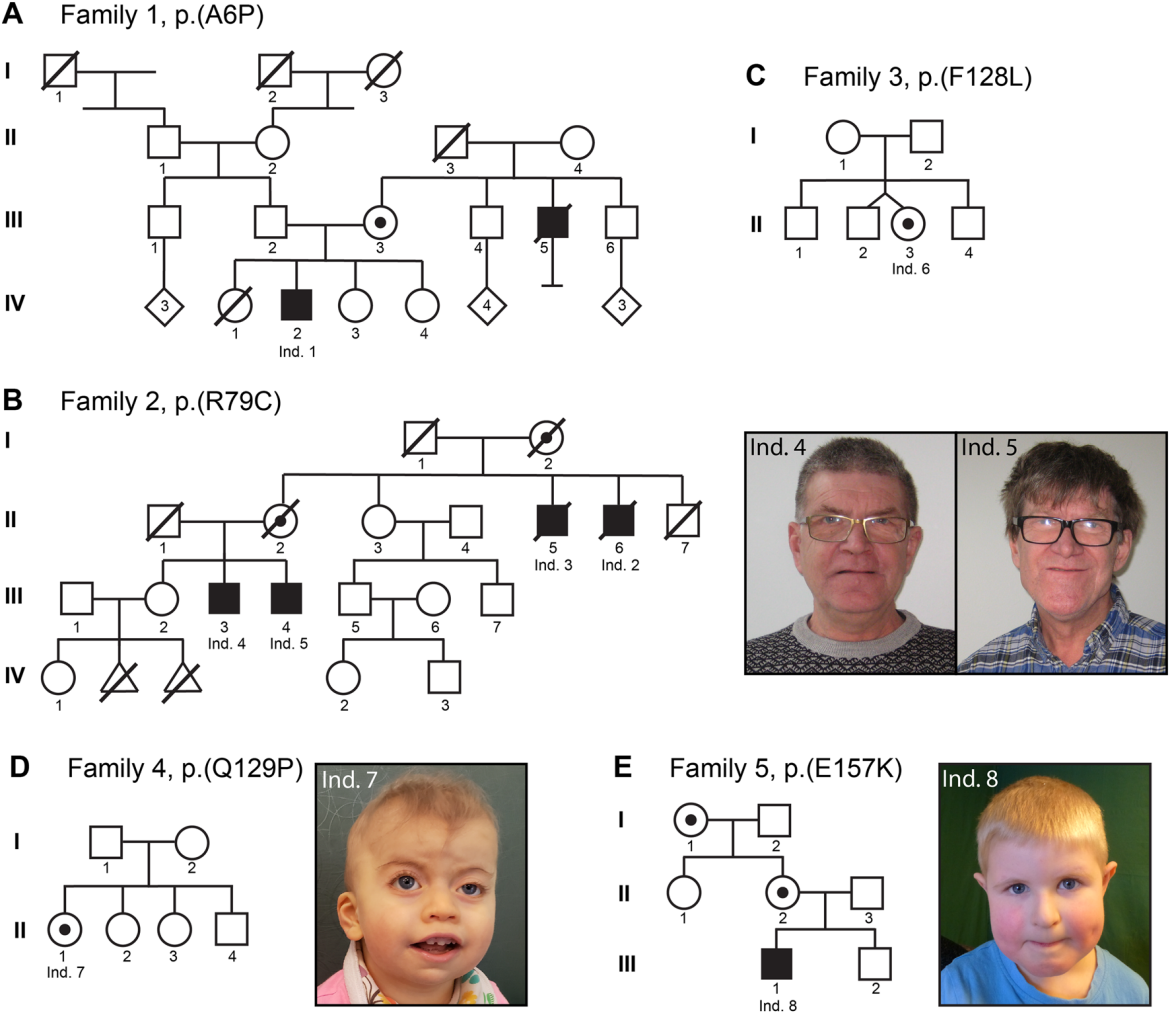
Family pedigrees and photographs. **A** Pedigree illustration for family 1. The male proband (Individual 1; F1:IV-2) displaying intellectual disability (ID) and cardiomyopathy harbours a c.16G>C p.(A6P) variant which was maternally inherited. A maternal uncle (F1:III-5) had ID and cardiomyopathy and died at age 40, but was never genetically tested. **B** Pedigree illustration for family 2. Three males (Individual 3–5; F2:II-5, F2:III-3 and F2:III-4) with ID were found to carry a maternally inherited c.235C>T p.(R79C) variant. Photographs show individual 4 and 5. A fourth male (Individual 2; F2:II-6) was not genetically tested, but displayed a similar phenotype and was a suspected carrier of the variant. **C** Pedigree illustration for family 3. The female proband (Individual 6; F3:II-3) has a de novo c.384T>G p.(F128L) variant. She has ID, microcephaly and central vision impairment. **D** Pedigree illustration for family 4. Photograph shows the female proband (Individual 7; F4:II-1) found to be heterozygous for a de novo c.386A>C p.(Q129P) variant. She has facial dysmorphism, microcephaly and cardiac anomalies. **E** Pedigree illustration for family 5. Photograph shows the male proband (Individual 8; F5:III-1) with a maternally inherited c.469G>A p.(E157K) variant. He displays autistic features, facial dysmorphism and microcephaly. *Ind.* individual

Individual 1 (F1:IV-2) is a 19-year-old man hemizygous for a novel *NAA10* c.16G>C p.(A6P) variant (Fig. 1A; Table 1). He has ID and hypertrophic cardiomyopathy with a family history suggesting an X-linked cause. The proband had a sister (F1:IV-1) who was born with an interrupted aortic arch and died at 10 days of life. Both of his living sisters and parents are healthy and had normal regular echocardiograms. A maternal uncle (F1:III-5) had ID, medullary sponge kidneys with renal failure, and died at age 40 from complications of an idiopathic restrictive cardiomyopathy. No one else in the family has been found to have medullary sponge kidneys or recurrent kidney stones, so this is not likely to be caused by *NAA10* impairment. There were concerns about the proband’s development from about 15 months of age. His mother reported that he was saying “mama” and “dada” at that age but stopped and did not start using words again until almost age 4 years. There were no concerns about his early motor development. He had issues with attention, concentration, and could be oppositional with his parents, teachers, and providers. He received special education all through school. Formal neuropsychological testing was attempted a number of times but without completion due to a lack of cooperation and effort. The neuropsychologist provided an estimated IQ < 60 based on what he was able to complete. At the completion of high school, he was reading at a 2nd-grade level and doing math at a 3rd-grade level.

**Table 1:** Summary of clinical findings in eight affected individuals with *NAA10* missense variants

Family	1	2	2	2	2	3	4	5
Individual	1	2	3	4	5	6	7	8
Gender	Male	Male	Male	Male	Male	Female	Female	Male
Age at most recent examination	19 years	52 years	37 years	24 years	22 years	16 years	33 months	8 years
Ethnicity	Filipino, European	Northern European	Northern European	Northern European	Northern European	White, non-Hispanic	White, non-Hispanic	Northern European
*NAA10* variants NC_000023.10 NM_003491.4	g.153200342C > G c.16G>C p.(Ala6Pro)	Not tested	g.153197875G>A c.235C>T p.(Arg79Cys)	g.153197875G>A c.235C>T p.(Arg79Cys)	g.153197875G>A c.235C>T p.(Arg79Cys)	g.153197526A>C c.384T>G p.(Phe128Leu)	g.153197524T>G c.386A>C p.(Gln129Pro)	g.153196218C>T c.469G>A p.(Glu157Lys)
Variant type	Hemizygous, maternal inherited	N/A	Hemizygous, maternal inherited	Hemizygous, maternal inherited	Hemizygous, maternal inherited	Heterozygous, de novo (parents tested and were normal)	Heterozygous, de novo (parents tested and were normal)	Hemizygous, maternal inherited
Body height and weight	168 cm (10–25th centile), 52.7 kg (3rd centile)	Age 52 years: 178 cm (50th centile), 68 kg (25–50th centile)	Small of posture	Small of posture	Small of posture	Age 15 years: 133.4 cm (< 2nd centile), 31.3 kg (< 2nd centile)	Age 32 months: 78.1 cm (< 2nd centile), 10.5 kg (1st centile)	At birth: 3.9 kg (66th centile), 53 cm (85th centile) Age 6 years: 22 kg (49th centile), 120 cm (55th centile)
Head circumference	56.8 cm (50–75th centile)	Assessed normal	Assessed normal	Assessed normal	Assessed normal	Age 15 years: 48.2 cm (< 2nd centile)	Age 36 months: 44.5 cm (< 2nd centile)	At birth: 34.5 cm (28th centile) Age 6 years: 48.5 cm (< 1st centile)
Facial and other dysmorphisms	Not dysmorphic. Broad nasal tip and thickened alae appropriate for family	Not dysmorphic. Oblonged face, with a narrow palate. Full lips	Not dysmorphic. Oblonged face, with a narrow palate	Not dysmorphic	Not dysmorphic	Not dysmorphic	Upper lid ptosis; prominent globes, short columella; flared nares; Bilateral “Sydney lines”	Round face, full cheeks, narrow palpebral fissures, epicanthal folds, smooth philtrum, simian crease, camptodactyly 4th fingers
Ambulation	Normal motor development and ambulation into adulthood	Able to walk	Walked at age 3 years	Sat at 9 months, walked at 23 months	Walked at age 10–12 months Low muscle tonus	Walked with a “walker” at age 6 years	Using a “stander” at age 33 months; not walking independently	Walked at age 21 months
Speech development	First words beyond “mama” at 4 years	First word at 3 years of age	Could say a few words at 4 years of age	Are speaking single words at 7 years of age	First word at 2 years, very low vocabulary at age 17	Non-verbal at age 17 years	Non-verbal at age 33 months	First words at age 12 months, but no sentences before age 3 years
Neurodevelopmental	Has intellectual disability but unwilling to cooperate with formal testing so FSIQ has been estimated at 60. Reading at 2nd grade and math at 3rd grade level at end of high school	Intellectual disability, IQ assessed to 19 at age 28 Nearsighted	Intellectual disability, IQ assessed to 31 at 10 years of age Normal sight and hearing as an adult. Concentration difficulties	Intellectual disabled, IQ assessed to 43 at age 14 Concentration difficulties. Hypermetropia	Intellecutual disability, IQ assessed to 56 at age 12. Concentration difficulties	Cerebral palsy; intellectual disability	Global developmental delay at age 33 months	Delayed development. Cognitive level lower normal. Autistic features
Seizures	No	Grand mal seizure, but EEG showed no abnormalities	No	No. EEG performed at 7 years of age, showed moderate abnormal	No	None documented (EEG normal)	No	No
Cardio	Hypertrophic cardiomyopathy with asymmetric septal hypertrophy. No arrhythmias	N/A	N/A	N/A	N/A	Normal echocardiogram and ECG (age 15 years)	Large atrial septal defect; “Ebsteinoid” tricuspid valve; left superior vena cava into coronary sinus torsades des points, SVT	ECG and cardiac ultrasound normal at age 7
Other	Maternal uncle deceased with intellectual disability and restrictive cardiomyopathy (not genetically tested)	Died at 52 years. Autopsy shows cerebral haemorrhage in the right remisphere and a tumour causing cortical compression			Has dysphagia, and had pyloric stenosis at an early age	46, XX karyotype MRI: cerebral parenchymal atrophy	47, XXX karyotype; MRI: cerebellar vermian hypoplasia; cystic dilatation of the 4th ventricle. Feeding difficulties requiring a G-tube	Hypermobile finger joints; Hypermetropia; Frowning face when excited

Hypertrophic cardiomyopathy was diagnosed at age 7 years. Regular follow-up since that time has confirmed persistent and stable asymmetric septal hypertrophy, most recently with a thickness of 18 mm without obstruction. He had no issues with fatigue, shortness of breath, chest pain, syncope, nor near syncope.

On physical examination at age 19 he had a height of 168 cm (10–25th percentile), weight of 52.7 kg (3rd percentile), and head circumference of 56.8 cm (50–75th percentile). He had bilateral indentations in the region of the lambdoid sutures. His nasal tip was broad with thickened alae. He had no additional facial dysmorphic features. His skin was normal without excessively laxity. He had normal muscle strength and tonus.

Previous clinical genetic testing included fragile-X testing, chromosome microarray, and an 18-gene hypertrophic cardiomyopathy gene panel. Furthermore, a brain magnetic resonance imaging (MRI) was normal.

Based on the family history suggesting an X-linked cause for ID and cardiomyopathy, genetic testing with a 116-gene next-generation sequencing panel for X-linked ID was carried out (Fulgent Genetics, Temple City, CA, USA) which identified the *NAA10* variant. Subsequent genetic testing of the proband’s mother (F1:III-3) confirmed that the variant was maternally inherited.

### Clinical report: family 2, individuals 2–5

Family 2 is a four-generation family with ID including four affected males across two generations (Fig. 1B; Table 1). Genetic testing was carried out in three individuals [individual 3 (F2:II-5), individual 4 (F2:III-3), and individual 5 (F2:III-4)], as the fourth individual 2 (F2:II-6) died before DNA was sampled. A novel *NAA10* c.235C>T p.(R79C) variant was detected in all three family members by exome sequencing with a special focus on the X-chromosome due to the X-linked inheritance pattern. The female carriers in this family were asymptomatic.

Individual 2 (F2:II-6) died at 52 years of age. He was diagnosed with moderate degree of ID and high degree myopia (− 10). IQ testing revealed an IQ of 17 at 23 years of age and an IQ of 19 at age 28. Height measured at age 52 was 178 cm (50th percentile) and weight was 68 kg (25–50th percentile). Physical findings included full lips and high vaulted palate. Mobility and muscle strength were normal, but lively patellar reflexes were noted. He developed some speech, but it remained very incomprehensive. Expressive aphasia was suspected as he understood most of what was said to him. He had a friendly personality. Hearing was estimated to be normal, but he was unable to cooperate with formal audiological testing. At age 48, he suffered a grand mal seizure but a subsequent EEG did not reveal any abnormalities. Two weeks prior to his death, he developed hyperreflexia, athetosis and tremor. Autopsy showed cerebral haemorrhage in the right hemisphere and a tumour causing cortical compression. The tumour was very cell-rich and with partial degeneration and felt to be metastatic, but a primary tumour was not identified. The cortex and white matter were atrophic. A single cavernous haemangioma was identified at autopsy in the liver. Genetic testing was not carried out, but due to his medical and family history, it is likely that he also had the same familial variant.

Individual 3 (F2:II-5), the younger brother of individual 2, was a male born naturally with a weight of approximately 3500 g (50th percentile). At age 2, he still had no verbal language and at age 3, he started walking independently. He never attended school and had an IQ of 31 at age 10. His language was very limited, but his speech comprehension was good. His hearing was determined normal at age 37. He had tremor and restlessness and was treated with chlorprothixene. He was mentally regarded as having moderate degree of ID. He was able to feed and toilet independently. No striking dysmorphic facial features were noted except a high vaulted palate. He died at around 51 years of life. No autopsy was performed.

Individual 4 (F2:III-3) is a 66-year-old male. He sat unsupported at age 9 months and walked at age 23 months. He had delayed speech development with elements of aphasia but better speech comprehension. He has received speech therapy for a long time. His hearing is estimated to be normal. He is hyperactive and restless by nature but easy going in contact with others.

At age 14, his IQ was 43. He has small stature, hypermetropia, but no facial dysmorphic features. At age 24, a chromosome analysis revealed normal male 46, XY karyotype. He currently lives in a protected living setting.

Individual 5 (F2:III-4) is a 64-year-old male. As an infant, he required surgery for pyloric stenosis as well as umbilical and inguinal hernia. He started to walk at 10–12 months and had some speech from age 2. At age 12, his IQ was 56. He had delayed speech development (dysphasia/expressive aphasia), but has benefitted from speech therapy and has obtained useful verbal competence for communication and exhibits good social skills. Chromosome analysis conducted at age 22 showed normal male 46, XY karyotype. As an adult, he is married and lives with his wife in an apartment in a protected living setting.

Previous clinical genetic testing included fragile-X testing, chromosome microarray, and a screening for known microdeletions and duplications by MLPA analysis [MLPA p106 X-linked ID syndromes and MLPA p245 Microdeletion Syndromes-1 (MRC Holland)] of Individual 5 (F2:III-4). The *NAA10* variant of the family was identified with exome sequencing as part of a research project to identify the underlying genetic factor in X-linked ID.

### Clinical report: family 3, individual 6

Individual 6 is a 17-year-old female who was found to be heterozygous for the previously studied pathogenic *NAA10* c.384T>G p.(F128L) variant (Saunier et al. 2016; Cheng et al. 2019) (Fig. 1C; Table 1). She was born at 34 weeks of gestation, with birth weight 2500 g (75th percentile). She was number one of fraternal twins. Her twin is healthy, with no health issues. In addition, she has two other healthy brothers. Early issues included respiratory distress, requiring supplemental oxygen for about 6 weeks. She was subsequently noted to have global DD and multiple contractures, including elbows and feet. There have been some issues with poor weight gain. She has central vision impairment, microcephaly and occasional tremors. She can walk with assistance but is frequently in a wheelchair. She was still incontinent at age 15 years. She was followed by paediatric cardiologists, and ECG and echocardiogram were unremarkable. At age 15 years, weight was 29.4 kg (< 3rd percentile, − 3 SD), height was 133.6 cm (< 3rd percentile, − 3 SD), and head circumference 48.2 cm (< 3rd percentile, − 3 SD). Findings at that time included contractures at the major joints, spasticity, and dysconjugate gaze. Laboratory evaluation included testing for Prader–Willi (OMIM #176270) and Angelman (OMIM #105830) syndromes, karyotyping, serum acylcarnitine profile and array CGH (comparative genome hybridisation), all of which were normal. The de novo* NAA10* variant was identified using clinical exome sequencing.

### Clinical report: family 4, individual 7

Individual 7 (F4:II-1) is a 33-month-old girl with a novel de novo* NAA10* c.386A>C p.(Q129P) variant (Fig. 1D; Table 1). She was born at 41 weeks of gestation with birth weight of 2910 g (23rd percentile) and birth length of 47 cm (12th percentile). She had perinatal distress requiring resuscitation. Apgar scores were 1, 3, and 9. Prenatal MRI showed hypoplastic cerebellar vermis and hypoplastic pons. Subsequent imaging after birth showed cerebellar vermian hypoplasia with cystic dilatation of the 4th ventricle. She also had a left pneumothorax. A heart murmur was noted, and she was found to have a small patent ductus arteriosus, enlarged atrial septal defect and Ebstein anomaly of the tricuspid valve. Bilateral vocal cord paralysis was noted. She had nutritional issues which required feeding with first gastrostomy tube and subsequently with a gastro-jejunostomy tube. Physical findings at 33 months included a bowed upper lip, thickened gingiva, upper lid ptosis, prominent globes, a short columella, microcephaly, bilateral Sydney lines, and a heart murmur. At age 33 months, she “babbles” but has no words. She can use a standing device but is not walking independently. A chromosomal microarray showed a 47, XXX karyotype, but as this finding could not explain her clinical picture, exome sequencing was carried out detecting the *NAA10* variant on one of her X chromosomes.

### Clinical report: family 5, individual 8

Individual 8 (F5:III-1) has a novel, hemizygous *NAA10* c.469G>A p.(E157K) variant (Fig. 1E; Table 1). He is the first child of healthy, unrelated parents and has one younger, healthy brother (III-2). The pregnancy and birth were uneventful and he had a normal head circumference at birth (34.5 cm, − 0.59 SD). Delayed psychomotor development was noted at age 1 year. He walked at age 21 months. Single words were spoken at age 1 year, but he did not put words together to form sentences until the age of 3. He displays autistic features like challenging interaction with other children, rigid behaviour, and loves sorting and systems. When excited, facial grimacing is observed. Mainstream school was started 1 year postponed. Formal testing at age 5 resulted in a diagnosis of a mixed specific developmental disorder with a cognitive level in the lower normal range. Muscular hypotonia was noted. His head circumference increased at a slow rate with decline to − 1.57 SD (46 cm) by 1 year of life and microcephaly by age 6 (− 2.67 SD, 48.5 cm). No cerebral MRI has been performed. He has a round face with full cheeks, narrow palpebral fissures, epicanthal folds, a smooth philtrum, bilateral single transverse palmar creases, and camptodactyly of the fourth fingers. The other finger joints are hypermobile. ECG and echocardiogram were performed at age 7 with normal results.

Genetic analyses including fragile-X testing and SNP array were normal. Diagnostic gene panel sequencing in a trio with his parents detected a *NAA10* variant inherited from his mother (F5:II-2). Segregation studies in the family show that the variant was inherited from his healthy maternal grandmother (F5:I-1) to his mother (F5:II-2). His healthy brother (F5:III-2) does not carry the variant. His mother (F5:II-2) is healthy, and no abnormalities were noted in an ECG performed following the gene test.

### NAA10 sequence conservation and structure

**Fig. 2:**
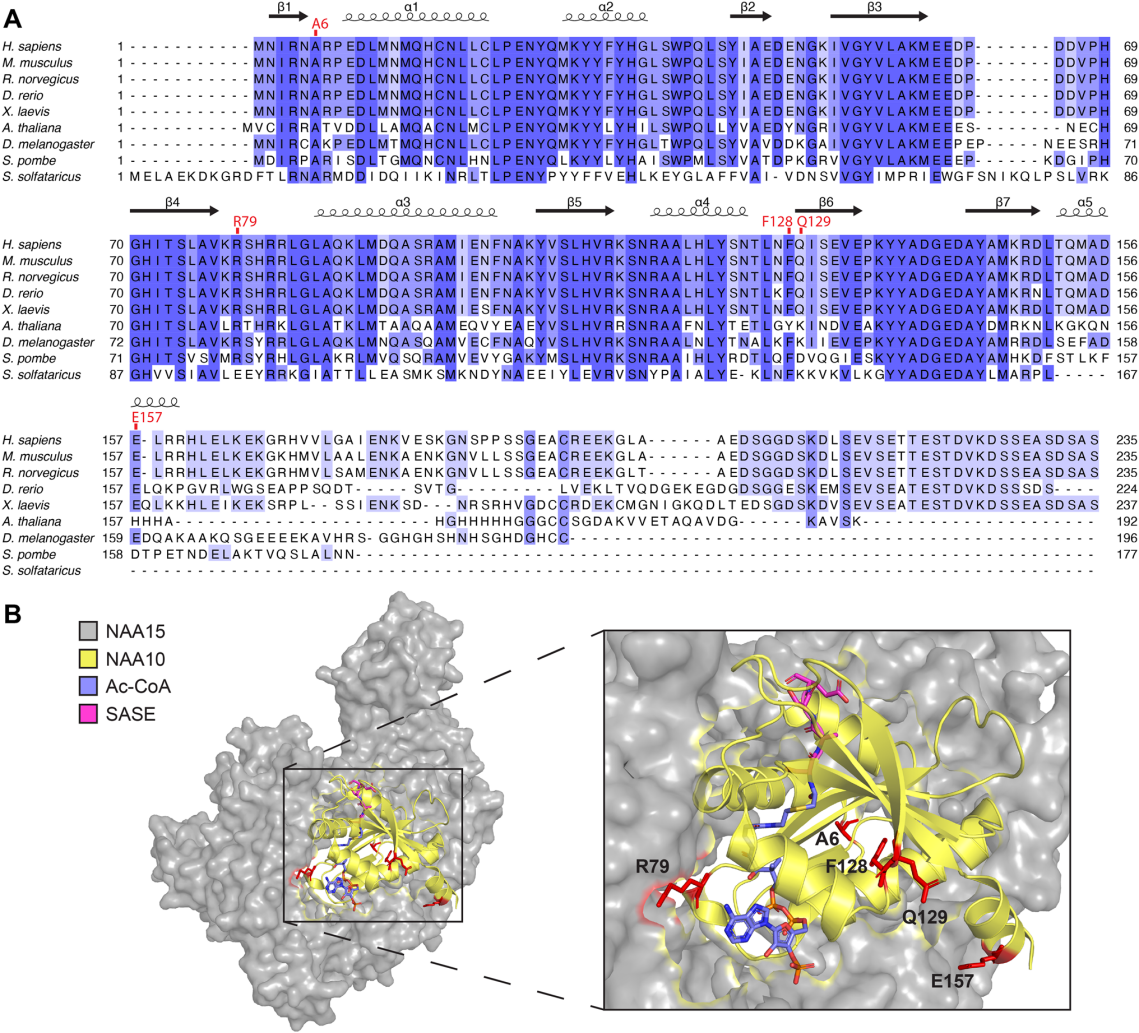
NAA10 sequence conservation, NatA structure and substituted residues. **A** Multiple sequence alignment displaying NAA10 amino acid conservation (indicated by a blue gradient) across nine species. NAA10 variant sites are indicated in red text above the alignment. Secondary structures were derived from human NatA (PDB ID: 6C9M). **B** Human NatA crystal structure (PDB ID: 6C9M) with NAA15 (grey) and NAA10 (yellow). The positions of NAA10 substitutions are highlighted in red. Acetyl coenzyme A (Ac-CoA) (blue) and serine–alanine–serine–glutamate-starting peptide (SASE) (magenta) in the active site were embedded in the structure from *S. pombe* NatA (PDB ID: 4KVM)

The evolutionary conservation of the five substituted NAA10 amino acids was investigated with multiple sequence alignment including nine species. NAA10 A6, R79 and F128 showed high conservation from yeast to human, whereas Q129 and E157 were mainly conserved in the animal kingdom (Fig. 2A). The high degree of conservation suggests an important role in maintaining NAA10 function. A three-dimensional NatA crystal structure (PDB ID: 6C9M) showed that A6 was located in the loop between β1 and α1, which is part of the NAA15 interaction domain (Fig. 2B). R79 was positioned close to the Ac-CoA-binding site; however, its side chain protruded towards NAA15 and was bioinformatically predicted to interact with several NAA15 residues (Fig. 2B). The missense variants p.(A6P) and p.(R79C) both affect amino acids situated near the NAA10–NAA15 interface and could potentially influence NatA complex formation and/or function. F128 was inward-pointing and part of a hydrophobic pocket at the core of NAA10. The side chains of Q129 and E157 were directed outwards of the NAA10 structure. Q129 was located at the start of β6, while E157 was located in α5 preceding the unstructured C-terminal tail. F128, Q129 and E157 were not located near the NAA10–NAA15-binding surface which suggests variants affecting these residues are more likely to impede monomeric NAA10 activity or stability rather than affecting the NatA complex itself.

### Cellular stability of *NAA10* variants

**Fig. 3:**
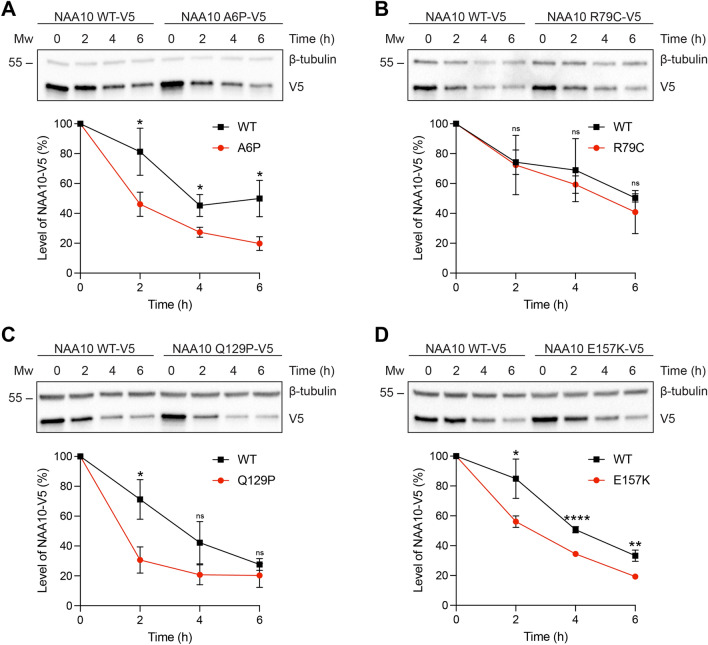
Cellular stability analysis of *NAA10* variants. HeLa cells transfected with NAA10 WT-V5, NAA10 A6P-V5 (**A**), NAA10 R79C-V5 (**B**), NAA10 Q129P-V5 (**C**), or NAA10 E157K-V5 (**D**) were treated with cycloheximide (CHX, 50 µg/ml) for 2–6 h and cell lysates were analysed by Western blot. Top panels in **A**–**D**: Western blot analysis of a CHX time course assay. Bottom panels in **A**–**D**: Stability curve showing the percentage level of NAA10-V5 at time points 2–6 h relative to the amount present at 0 h and β-tubulin as a loading control. Each stability curve shows the mean ± SD of three independent experiments performed per *NAA10* variant. Significance was calculated by a two-tailed Student’s *t *test. ****P ≤ 0.0001; **P ≤ 0.01; *P ≤ 0.05; *ns* not significant *P* > 0.05

In order to investigate the cellular stability of the novel *NAA10* missense variants, cycloheximide (CHX) chase assays were conducted in transfected HeLa cells. NAA10 A6P-V5, NAA10 Q129P-V5 and NAA10 E157K-V5 revealed a reduced stability compared to NAA10 WT-V5 throughout a 6-h time course (Fig. 3A, C, D). In contrast, NAA10 R79C-V5 displayed a similar turnover rate as for NAA10 WT-V5, indicating that this variant does not impact NAA10 stability (Fig. 3B).

### NatA complex formation and catalytic activity

**Fig. 4:**
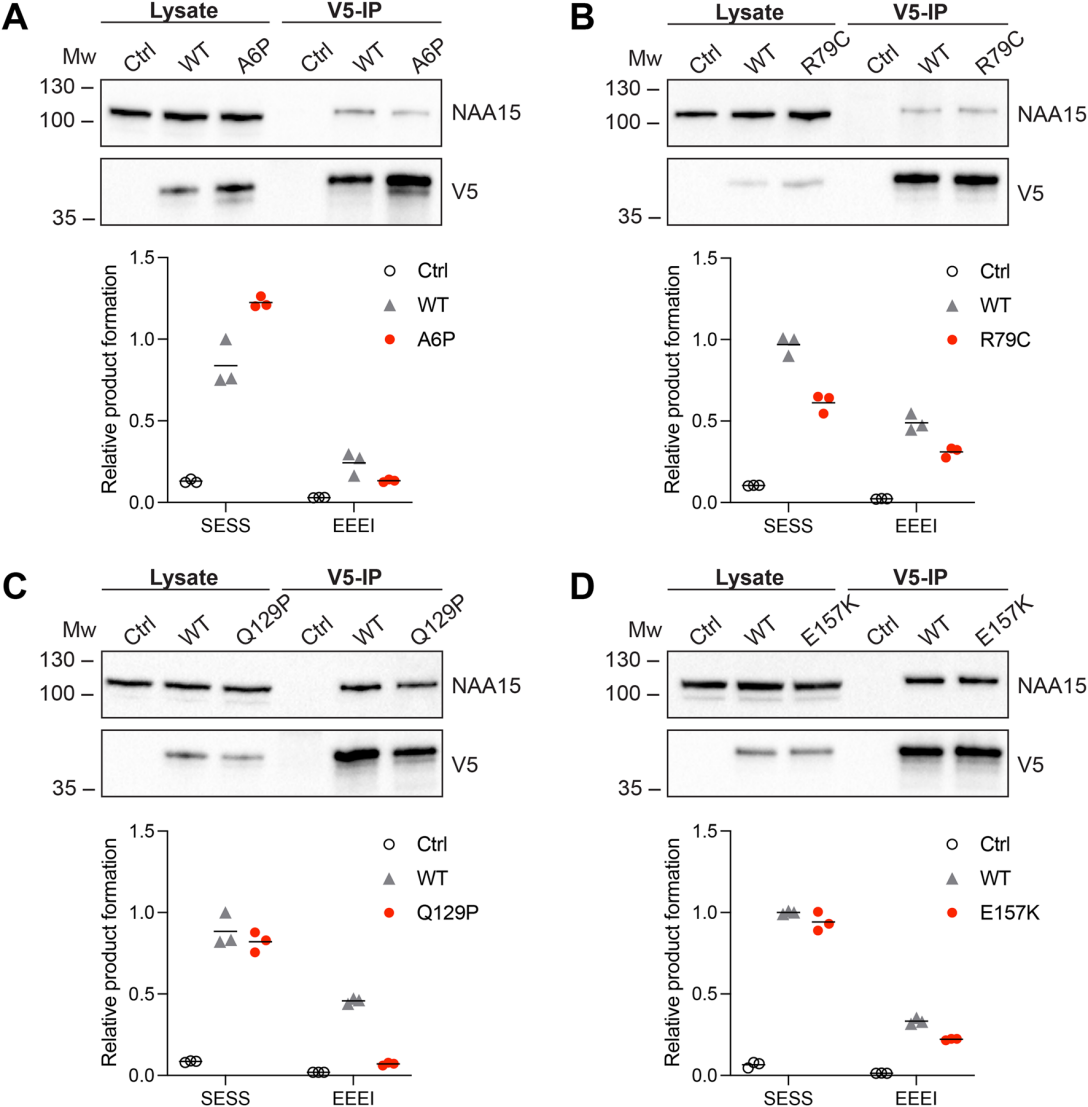
NatA complex formation and N-terminal acetylation by *NAA10* variants. Top panels in **A**–**D**: Western blot analysis of V5-immunoprecipiation from HeLa cells overexpressing NAA10 WT-V5 or the variants NAA10 A6P-V5 (**A**), NAA10 R79C-V5 (**B**), NAA10 Q129P-V5 (**C**), or NAA10 E157K-V5 (**D**). Bottom panels in **A**–**D**: Immuno-precipitated NAA10 WT or *NAA10* variants were comparatively tested in Nt-acetylation assays using the NatA substrate SESS and monomeric NAA10 in vitro substrate EEEI. β-gal-V5 pull-down was used as input in negative control reactions. The values for Nt-acetylated SESS and EEEI product formation were normalised to the band intensities of NAA15 and NAA10, respectively, and shown as relative to WT. The experiments were performed in at least three independent setups for each *NAA10* variant (Fig. S1). One representative experiment with technical triplicates is shown. The mean of the triplicates is indicated by a black line

NatA complex formation with NAA15 of the *NAA10* variants was investigated in HeLa cells by co-immunoprecipitation experiments. This revealed that fivefold less NAA15 co-immuno-precipitated with NAA10 A6P-V5 compared to NAA10 WT-V5, suggesting that *NAA10* p.(A6P) is highly reduced in its capacity to form the NatA complex (Fig. 4A). On the other hand, there was no significant difference in co-immunoprecipitation of NAA15 with NAA10 WT-V5 and the three variants NAA10 R79C-V5, NAA10 Q129P-V5 and NAA10 E157K-V5 (Fig. 4B–D). Thus, these three *NAA10* variants seemingly do not impair NAA10–NAA15 binding. The immuno-precipitated NAA10-V5 were further subjected to Nt-acetylation assays in order to investigate the catalytic activity of the different *NAA10* variants. A serine–glutamate–serine–serine-starting peptide (SESS) derived from a canonical NatA substrate was used to test NatA activity and, since monomeric NAA10 has a high preference for acidic N-termini in vitro, a glutamate–glutamate–glutamate–isoleucine-starting peptide (EEEI) was used to measure monomeric NAA10 NAT activity (Arnesen et al. 2009; Damme et al. 2011). The measured product formation of Nt-acetylated SESS and EEEI was normalised to the amount of NAA15 (as a measure of NatA complex) and NAA10 present in the reaction, respectively. The only variant exhibiting reduced NatA catalytic activity after normalisation was NAA10 R79C-V5 (Fig. 4B). Moreover, all the four *NAA10* variants tested displayed reduced Nt-Ac of the EEEI peptide as compared to WT, suggesting the monomeric NAA10 NAT function is attenuated (Fig. 4A–D). Notably, NAA10 Q129P-V5 had an almost abolished catalytic activity towards EEEI, whereas A6P, R79C and E157K displayed a more moderate reduction.

## Discussion

In this study, we have carried out biochemical characterisation of four novel *NAA10* missense variants [p.(A6P), p.(R79C), p.(Q129P) and p.(E157K)] to evaluate their pathogenicity, and clinically described one new individual with a p.(F128L) variant. All the variants are likely to affect the stability, function and/or complex formation of NAA10.

NAA10 Nt-acetylates nearly half of the human proteome as the catalytic subunit of the NatA complex together with the large auxiliary subunit NAA15 (Arnesen et al. 2009). Thus, it was of interest to determine whether the *NAA10* variants affected the binding affinity towards NAA15. Based on the bioinformatic analysis, we postulated that the *NAA10* p.(A6P) variant might hamper NatA complex formation as a consequence of its location near the NAA15-binding domain (Fig. 2), and this was supported by the biochemical evidence. NAA10 A6P-V5 co-precipitated less NAA15 compared to NAA10 WT-V5, and thus displayed a decreased capacity to form NatA complexes (Fig. 4A). Notably, the portion of NAA10 A6P-V5 that was bound to NAA15 appeared to be functional in Nt-acetylating NatA substrates, while Nt-Ac of the monomeric NAA10 in vitro substrate EEEI was reduced. However, the overall Nt-Ac of NatA substrates in cells expressing p.(A6P) may also be decreased due to its instability (Fig. 3A) and less NatA complex being formed. p.(A6P) thereby resembles previously studied missense variants in the N-terminus of NAA10, p.(D10G), p.(L11R) and p.(H16P), which also hampered NatA complex formation (Bader et al. 2020; McTiernan et al. 2020). However, it is worth noting that p.(L11R) and p.(H16P) were identified in females only and the male harbouring p.(D10G) died during infancy; hence, the p.(A6P) variant presented here in a 19-year-old male appears to be less severe.

Although R79 was bioinformatically predicted to interact with NAA15 residues, NAA10 R79C-V5 was not perturbed in its NatA complex formation capacity (Fig. 4B) potentially because R79 is not essential for NAA15 binding or C79 is able to form compensating interactions. NAA10 R79C-V5 exhibited decreased Nt-Ac of both the NatA substrate SESS and monomeric NAA10 in vitro substrate EEEI (Fig. 4B). The suboptimal Nt-Ac of substrates may be caused by structural changes in the active site since the p.(R79C) variant is close to the Ac-CoA-binding pocket (Fig. 2B). Remarkably, the family members with p.(R79C) represent the oldest living males with a pathogenic *NAA10* variant reported to date. The most prominent phenotype of these probands was ID, although an extensive medical history was not available. The absence of clinical evidence of cardiac problems may partially explain the milder phenotype and longer survival. There is at present no simplistic explanation as to why male carriers of some variants, such as p.(R79C), can live to adulthood, whereas other variants are fatal during infancy.

The p.(Q129P) variant affects Q129 located at the start of β6, and the substitution with proline is likely to shorten the β-strand (Fig. 2). As the β6-β7 loop is considered important for substrate binding of NAA10 (Liszczak et al. 2013), the p.(Q129P) variant may introduce alterations to the active site that could affect NAA10 function. Our functional studies showed that p.(Q129P) did not hamper NatA complex formation (Fig. 4C), which was as expected since the variant site is not located near the NAA15-binding interface. Correspondingly, the NatA Nt-Ac activity remained intact, while the monomeric NAA10 NAT activity was nearly abolished (Fig. 4C). Moreover, NAA10 Q129P-V5 was highly destabilised after 2 h of CHX treatment (Fig. 3C). These results imply that p.(Q129P) impacts NAA10 stability and monomeric NAT function, and the underlying disease mechanism is likely related to NAA10’s monomeric modes of action rather than NatA-mediated Nt-Ac. p.(Q129P) has a similar biochemical profile as the most frequently reported NAA10 variant, p.(R83C), and the p.(Q129P) proband exhibits many phenotypes observed in females with the p.(R83C) variant (Saunier et al. 2016; Maini et al. 2021). It should, however, be noted that the female presented herein has a 47, XXX karyotype and it remains unclear to which degree the clinical features can be attributed to this chromosome disorder.

Similarly, NAA10 E157K-V5 did not debilitate NAA15 binding nor NatA-mediated Nt-Ac (Fig. 4D). Nt-Ac of the acidic substrate EEEI was decreased, suggesting that the monomeric NAA10 NAT activity was attenuated. The variant also caused destabilisation of the NAA10 protein (Fig. 3D), potentially due to altered electrostatic properties in this position. Notably, E157 is located in the α5 helix near the flexible and less conserved C-terminal tail of NAA10 (Fig. 2). The functionality of the C-terminus of NAA10 is not clear, but it contains multiple phosphorylation sites which may confer various modes of regulation (Målen et al. 2009). Since p.(E157K) seemingly affects monomeric NAA10 and not the NatA complex, we speculate that the NAA10 C-terminus may be more important for the monomeric roles of NAA10. In support of this, the C-terminal tail is not conserved in, for instance, yeast, where a monomeric role of NAA10 has not yet been presented (Fig. 2). When compared to p.(D10G) and p.(S37P), which had fatal consequences in males (Rope et al. 2011; Cheng et al. 2019), the p.(E157K) variant represents another case of less severe *NAA10*-related syndrome in males. It is possible that the milder phenotype is partially due to the fact that the major pool of NatA substrates is likely not to be affected by this variant.

The female harbouring a de novo p.(F128L) variant has DD, microcephaly and central visual impairment and thus has phenotypic similarities to other females reported with this variant (Saunier et al. 2016; Cheng et al. 2019). Visual impairments, such as central or cortical visual impairment, astigmatism or strabismus, appear to be a frequent phenotype occurring in half of all individuals with *NAA10* missense variants (Supplementary Table S1). However, only polyadenylation signal (PAS) variants, splice and frameshift variants in *NAA10* have been found to cause microphthalmia (Cheng et al. 2019; Esmailpour et al. 2014; Johnston et al. 2019).

p.(F128L), p.(Q129P), and p.(E157K) affect nucleotides close to exon–intron boundaries in *NAA10* which potentially could affect mRNA splicing and contribute to the pathogenicity of these variants. However, in silico predictions using SpliceAI (https://spliceailookup.broadinstitute.org/) and VarSEAK (https://varseak.bio/) indicated that these variants do not affect mRNA splicing and this was therefore not investigated experimentally.

In the last decade, 21 *NAA10* missense variants have been reported as pathogenic in 69 males and females and can be classified as causative of *NAA10*-related syndrome. The majority of these variants have been biochemically characterised through enzymatic activity and/or stability assays [p.(D10G), p.(L11R), p.(H16P), p.(S37P), p.(Y43S), p.(I72T), p.(R83C), p.(R83H), p.(N101K), p.(A104D), p.(V107F), p.(V111G), p.(R116W), p.(F128L), p.(F128I), p.(M147T)], whereas others have only been described clinically [p.(A87S), p.(E100K), p.(L121V)] (Myklebust et al. 2015; Casey et al. 2015; Popp et al. 2015; Saunier et al. 2016; McTiernan et al. 2018, 2020, 2021; Støve et al. 2018; Cheng et al. 2019; Ree et al. 2019; Bader et al. 2020; Afrin et al. 2020). Furthermore, a frameshift variant, a splice-site variant and three PAS variants in *NAA10* have been associated with syndromic microphthalmia in 20 males (Cheng et al. 2019; Esmailpour et al. 2014; Johnston et al. 2019). A full overview of all *NAA10* variants reported can be found in Supplementary Table S1. Functional studies suggest that missense variants can affect distinct functions of NAA10. For instance, p.(L11R), p.(H16P) and p.(N101K) exclusively debilitate the NAT activity of NatA. In contrast, p.(I72T), p.(R83C), p.(V111G), as well as p.(Q129P) and p.(E157K) from our study, appear to solely attenuate monomeric NAA10 NAT activity. Moreover, many variants are hypomorphic and affect both of these functions, such as p.(A6P) and p.(R79C), presented herein (Supplementary Table S1) (Saunier et al. 2016; McTiernan et al. 2018, 2021; Støve et al. 2018; Cheng et al. 2019; Bader et al. 2020).

Additionally, variants may affect NAA10-mediated lysine acetylation or non-catalytic regulation which generally has not been addressed experimentally. The exception is one study which found that binding of NAA10 to imprinting control regions was decreased for three variants, p.(S37P), p.(V107F) and p.(R116W), potentially resulting in gene dysregulation (Lee et al. 2017).

The broad range of phenotypes observed in affected individuals is likely due to various downstream effects caused by distinct variants and how they may affect different roles of NAA10. Additionally, genetic variation between individuals is likely to contribute significantly to the clinical phenotypes. Consequently, the involvement of NAA10 in numerous cellular pathways and the resulting pleiotropic effects make it difficult to distinguish pathomechanisms. Thus, for males, the phenotypic spectrum is probably related to the above and to the level and type of protein dysfunctionality of a particular *NAA10* variant. For female carriers, X-chromosome skewing (degree of skewing, tissue specificity of skewing etc.) will additionally decide whether or not an individual will be healthy and the type and strength of clinical phenotypes.

Out of the five *NAA10* variants reported in this study, p.(E157K) was the only variant listed in Genome Aggregation Database (gnomAD) (https://gnomad.broadinstitute.org/), and this variant was only found in one hemizygote with no phenotypic information. If the p.(E157K) variant is found in a healthy male it would imply the variant is not disease-causing. Thus, the support for pathogenicity of this variant is considered weaker than for the other variants reported herein. The identification of other individuals with the p.(E157K) variant would help elucidate the pathogenicity of this variant.

In ClinVar (https://www.ncbi.nlm.nih.gov/clinvar/) (Landrum et al. 2018), the previously reported p.(F128L) variant was registered as pathogenic (VCV000280237.3), while p.(A6P) and p.(E157K) were not registered and p.(R79C) and p.(Q129P) were listed as variants of uncertain significance (VCV000391316.3 and VCV000843535.2). Based on the clinical information and biochemical characterisation ascertained in our study, p.(A6P), p.(R79C), and p.(Q129P) classify as pathogenic variants according to American College of Medical Genetics (ACMG) standards (Supplementary Table S2) (Richards et al. 2015). *NAA10* p.(E157K) classifies as likely pathogenic and further studies should be conducted to fully define causality.

In conclusion, we have reported phenotypic information on eight individuals from five families with five different inherited or de novo* NAA10* variants. Functional investigations of the four novel variants combined with data on the previously defined pathogenic *NAA10* variants provide support for at least two and possibly more distinct disease mechanisms. These are likely to be steered by different biochemical activities of NAA10 and a plethora of downstream signalling events may result in pleiotropic and overlapping phenotypes in the affected individuals. The continued discovery of novel *NAA10* variants and their functional impact is an important step towards a better understanding of the complex genotype–phenotype correlation in individuals with *NAA10*-related syndrome. This study also underlines the importance of functional studies to understand the pathogenicity of missense variants.

## Materials and methods

### Plasmids and antibodies

Plasmids were constructed to encode the four different *NAA10* (NM_003491.4) variants; *NAA10* c.16G>C p.(A6P), *NAA10* c.235C>T p.(R79C), *NAA10* c.386A>C p.(Q129P), and *NAA10* c.469G>A p.(E157K). The mutations were incorporated into a pcDNA3.1/*NAA10*-WT-V5 vector [previously called phARD1-V5 (Arnesen et al. 2005)] using Q5 Site Directed Mutagenesis Kit (New England Biolabs). The primers used for mutagenesis are available in Supplementary Table S3. The resulting plasmids were verified by Sanger sequencing. Primary antibodies used in this study were V5-tag mouse monoclonal antibody (1:5000 dilution, Invitrogen R960-25), β-tubulin mouse monoclonal antibody (1:3000 dilution, Sigma-Aldrich T5293) and NAA15 rabbit polyclonal antibody (1:2000, Biogenes, previously called anti-NATH) (Arnesen et al. 2005). Secondary antibodies used were Mouse IgG HRP-Linked Whole Ab (1:5000 dilution, Cytiva NA931) and Rabbit IgG HRP-Linked Whole Ab (1:5000 dilution, Cytiva NA934).

### Cell culture and transfection

HeLa cells (ATCC CCL-2) were grown in Dulbecco’s Modified Eagle’s medium (Merck) supplemented with 10% fetal bovine serum and 1% penicillin/streptomycin at 37 °C and 5% CO_2_. HeLa cells were transfected with plasmids using XtremeGENE9 DNA Transfection Reagent (Roche) and grown for 48 h before harvesting. For immunoprecipitation and Nt-acetylation assays, 2 × 10^6^ HeLa cells were seeded 16 h prior to transfection in five 10 cm dishes per sample. Cells were transfected with 4 µg of plasmid encoding *NAA10* WT-V5, 6–8 µg of plasmids encoding the *NAA10* variants or 6–8 µg of plasmid encoding *LacZ*-V5 as a negative control. Different amounts of plasmids were used in order to obtain apparently equal protein levels assessed by Western blotting. For cycloheximide chase assays, 3 × 10^5^ HeLa cells were seeded in 6-well plates and each well was transfected with 1.2 µg of *NAA10* WT-V5 plasmid or 1.8–2.4 µg of plasmids encoding the *NAA10* variants. *NAA10* WT-V5 was co-transfected with empty V5-plasmid to provide equal conditions.

### Cycloheximide chase assay

Cycloheximide chase assays were performed in order to compare the stability of the four *NAA10* variants with NAA10 WT-V5. 48 h post transfection, HeLa cells were treated with 50 µg/ml cycloheximide (Merck) and harvested 2, 4, or 6 h post treatment. Untreated cells were harvested at time point 0 h as a reference point. The amount of NAA10-V5 present at the different time points was quantified relative to the amount present at time point 0 h and normalised to the loading control β-tubulin. Harvested cells were lysed in 40 µl lysis buffer (50 mM Tris–HCl pH 8.0, 150 mM NaCl, 5 mM EDTA, 0.5% NP-40) and the cell extracts were analysed by Western blot to compare NAA10 turnover.

### Immunoprecipitation and Nt-acetylation assay

In order to investigate NatA complex formation and catalytic activity, NAA10-V5 was immuno-precipitated from transfected HeLa cells. Cells were harvested by scraping and centrifuged at 1500×*g* for 5 min. Pelleted cells were re-suspended in 1 ml lysis buffer (50 mM Tris–HCl pH 8.0, 150 mM NaCl, 5 mM EDTA, 0.5% NP-40) and incubated on a rotator at 4 °C for 10 min to lyse the cells. Cell lysates were then centrifuged at 17,000×*g* for 5 min and the supernatants collected. 4 µg of anti-V5-tag mouse monoclonal antibody (Invitrogen, R960-25) was added to the supernatants and incubated at 4 °C for 2–3 h on a rotator. 30 µl Dynabeads Protein G (Invitrogen) per sample was washed three times in lysis buffer, added to the cell lysates and incubated overnight at 4 °C on a rotator. Following overnight incubation, the magnetic beads were washed three times in lysis buffer before the beads were re-suspended in 95 µl acetylation buffer (50 mM Tris–HCl pH 8.5, 1 mM EDTA, 10% Glycerol). Nt-acetylation assays were performed as described previously (Drazic and Arnesen 2017). 25 µl reactions were run in triplicates and contained 10 µl immuno-precipitated enzyme, 50 µM [^14^C]-Ac-CoA (PerkinElmer), 200 µM oligopeptide SESS_24_ (SESSSKSRWGRPVGRRRRPVRVYP) or EEEI_24_ (EEEIAALRWGRPVGRRRRPVRVYP) (BioGenes), and acetylation buffer. Reactions without peptide or with control immunoprecipitation sample were used as negative controls. After incubating the reactions at 37 °C for 30 min on a thermoshaker, 23 µl of each sample was added to P81 phospho-cellulose filter discs (Millipore). Excess [^14^C]-Ac-CoA was removed by three washes in 10 mM HEPES buffer (pH 7.4). Dried filter discs were added to 5 ml Ultima Gold F scintillation mixture (PerkinElmer) and product formation was determined by a TriCarb 2900TR Liquid Scintillation Analyzer (PerkinElmer). In such in vitro acetylation assays, samples enriched in NAA10-V5 (and NAA15, if binding to NAA10 is maintained for *NAA10* variants) are used as enzyme input. Other co-immuno-precipitating proteins could theoretically impact the measured activity. This has not been specifically addressed in this study, but we have not detected any significant enrichment of other acetyltransferase enzymes in similar samples previously.

### Western blot analysis

Cell lysates or immunoprecipitation samples were mixed with 6X SDS sample buffer (Alfa Aesar) and boiled for 5 min at 95 °C. Samples were run through SDS-PAGE to separate proteins and transferred onto a nitrocellulose membrane using a standard Western blot protocol. Blots were blocked with 5% non-fat dry milk and incubated with primary antibodies overnight. After three washes with PBS-T, blots were incubated with secondary antibodies for 1 h. Blots were imaged using ChemiDoc XRS+ system (Bio-Rad) and Imagelab Software (Bio-Rad).

### Bioinformatic analysis

ClustalOmega (Sievers et al. 2011) was used to create a multiple sequence alignment of NAA10 sequences (Supplementary Table S4) and conservation was annotated by Jalview (Waterhouse et al. 2009). The hNatA structure (PDB ID: 6C9M) (Gottlieb and Marmorstein 2018) was superimposed with Ac-CoA and peptide SASE from *S. pombe* NatA (PDB ID: 4KVM) (Liszczak et al. 2013) and visualised using PyMOL v. 2.3.1 (Schrödinger).

### Exome sequencing

The *NAA10* variants were identified in a clinical diagnostic setting using exome sequencing. Briefly, genomic DNA from probands and/or parents were enriched for targeted regions using hybrid capture technology. Next-Generation Sequencing technology was then used to sequence prepared DNA libraries. Reads were aligned to the human genome reference sequence (GRCh37/hg19), and variants were detected in regions of at least 10× coverage. Data were subsequently annotated, filtered and analysed in order to identify sequence variants as well as large deletions and/or duplications. The detailed protocols and technology utilised varied based on the companies involved in the exome sequencing; Fulgent Genetics (Temple City, CA, USA) for family 1, Illumina (San Diego, CA, USA) for family 2 and 5, GeneDx (Gaithersburg, MD, USA) for family 3, and Invitae (San Francisco, CA, USA) for family 4.

## Supplementary Information

Below is the link to the electronic supplementary material.

Supplementary file1 (DOCX 5053 kb)

## Data Availability

The *NAA10* variants are available at ClinVar (https://www.ncbi.nlm.nih.gov/clinvar/): c.16G>C p.(A6P) accession number SCV001981511; c.235C>T p.(R79C) accession number SCV001980711; c.384T>G p.(F128L) accession number SCV001981509; c.386A>C p.(Q129P) accession number SCV001981510; c.469G>A p.(E157K) accession number SCV002032173. c.469G>A p.(E157K) can also be found at Decipher (http://www.deciphergenomics.org) with accession number 407850.

## Abbreviations

Ac-CoA: Acetyl coenzyme A
CHX: Cycloheximide
DD: Developmental delay
EEEI: Glutamate–glutamate–glutamate–isoleucine-starting peptide
ID: Intellectual disability
MRI: Magnetic resonance imaging
NAT: N-terminal acetyltransferase
Nt-Ac: N-terminal acetylation
PAS: Polyadenylation signal
SASE: Serine–alanine–serine–glutamate-starting peptide
SESS: Serine–glutamate–serine–serine-starting peptide
SD: Standard deviation
