# Supplementary material for: Biochemical analysis of novel NAA10 variants suggests distinct pathogenic mechanisms involving impaired protein N-terminal acetylation
Source: Hum Genet. 2022 Jan 17;141(8):1355–69. doi: 10.1007/s00439-021-02427-4 (PMC9304055; doi:10.1007/s00439-021-02427-4)
Supplement: Supplementary file 1 — Supplementary file1 (DOCX 5053 kb) [file 439_2021_2427_MOESM1_ESM.docx]

**Table S1. Overview of *NAA10* variants**

| **NAA10 variant** | **A6P** | **D10G** | **L11R** | **H16P** | **S37P** | **Y43S** | **I72T** | **R79C** | **R82Q^§^** | **R83C** | **R83H** | **A87S** | **E100K** |
| --- | --- | --- | --- | --- | --- | --- | --- | --- | --- | --- | --- | --- | --- |
| Publication | Present study | Cheng *et al.* 2019, McTiernan *et al. 2020* | Cheng *et al.* 2019, McTiernan *et al.* 2020 | Bader *et al.* 2020 | Rope *et al.* 2011, Myklebust *et al.* 2014,  Gogoll *et al.* 2021 | Casey *et al.* 2014, Cheng *et al.* 2019 | Støve *et al.* 2018,  Cheng *et al.* 2019 | Present study | DDDS *et al.* 2017 | Saunier *et al.* 2016, Sidhu *et al.* 2017, DDDS *et al.* 2017, Huang *et al.* 2018, Kim *et al.* 2019, Gupta *et al.* 2019, Cheng *et al.* 2019, Maini *et al.* 2021 | Ree *et al.* 2019 | Cheng *et al.* 2019 | Afrin *et al.* 2020 |
| NatA complex formation | Defect | Defect | Defect | Defect | Defect | NA | NA | Intact | NA | NA | NA | NA | Defect |
| NatA activity | Intact | Intact | Defect | Defect | Defect | Defect | Intact | Defect | NA | Intact | NA | NA | NA |
| NatA thermostability | NA | Defect | Defect | NA | Defect | Defect | Intact |  | NA | Defect | NA | NA | NA |
| NAA10 activity | Defect | Defect | Intact | Intact | Defect | Defect | Defect | Defect | NA | Defect | Defect | NA | NA |
| NAA10 cellular stability | Defect | NA | NA | Intact | NA | Defect | Defect | Intact | NA | Intact | NA | NA | NA |
| Individuals | 1 M | 1 M | 1 F | 1 F | 9 M | 2 M | 3 M | 4 M | 1 F | 25 F  1 M | 2 M | 3 F | 1 M  1 F |
| Number of individuals per variant displaying the following traits |  |  |  |  |  |  |  |  |  |  |  |  |  |
| Maternal inheritance | 1 |  | NA |  | 9 | 2 | 3 | 4 |  | 2 | 1 |  | 2 |
| *De novo* |  | 1 | NA | 1 |  |  |  |  | 1 | 23 | 1 | 3 |  |
| ID | 1 |  |  | 1 |  | 2 | 3 | 4 |  | 20 | 2 | 2 |  |
| DD | 1 | 1 | 1 | 1 | 6 |  | 3 |  | 1 | 25 | 2 | 3 | 2 |
| Muscular hypotonia |  | 1 |  |  | 8 | 2 |  |  |  | 12 | 1 | 2 |  |
| Speech delay | 1 |  | 1 | 1 |  |  | 3 | 4 |  | 19 | 2 | 3 | 1 |
| Feeding difficulties |  | 1 | 1 | 1 | 8 |  | 1 |  |  | 15 | 1 | 2 |  |
| Seizures |  |  |  |  | 3 | 1 |  | 1 |  | 9 | 1 | 2 |  |
| Brain imaging abnormalities |  | 1 | 1 | 1 | 6 | 2 | 2 |  |  | 11 |  | 2 | 1 |
| Behavioural abnormalities |  |  |  | 1 |  |  | 1 |  |  | 11 | 2 | 1 |  |
| Cardiac anomalies | 1 | 1 |  |  | 8 | 2 | 3 |  |  | 11 | 2 |  | 1 |
| Visual impairments |  |  | 1 | 1 | 2 | 1 | 1 | 2 |  | 14 |  | 1 |  |
| Microphthalmia |  |  |  |  |  |  |  |  |  |  |  |  |  |
| Hearing impairments |  | 1 |  | 1 | 1 |  |  |  |  | 3 |  | 1 |  |
| Skeletal abnormalities |  | 1 |  | 1 | 7 | 2 |  |  |  | 12 |  | 1 | 1 |
| Genitourinary abnormalities |  |  |  |  | 5 |  |  |  |  | 1 |  | 1 | 1 |
| Growth failure |  |  |  |  | 7 | 1 |  |  |  | 10 |  | 1 |  |
| Short stature |  |  |  | 1 | 5 |  |  |  |  | 2 |  | 1 |  |
| Microcephaly |  |  |  | 1 | 5 |  |  |  |  | 3 |  | 2 |  |
| Dysmorphic features |  | 1 |  | 1 | 8 | 2 | 1 | 2 |  | 14 | 1 | 2 | 1 |

**Table S1 continued.**

| **NAA10 variant** | **N101K** | **A104D** | **V107F** | **V111G** | **R116W** | **H120P^§^** | **L121V** | **F128L** | **F128I** | **Q129P** | **M147T** | **E157K** |
| --- | --- | --- | --- | --- | --- | --- | --- | --- | --- | --- | --- | --- |
| Publication | McTiernan *et al.* 2021 | Cheng *et al.* 2019 | Popp *et al.* 2015 | McTiernan *et al.* 2018 | Popp *et al.* 2015, Saunier *et al.* 2016, Valentine *et al.* 2018 | DDDS *et al.* 2017 | Cheng *et al.* 2019 | Saunier *et al.* 2016, DDDS *et al.* 2017, Cherot *et al.* 2018, Cheng *et al.* 2019,  Present study | Saunier *et al.* 2016 | Present study | Cheng *et al.* 2019 | Present study |
| NatA complex formation | Defect | NA | NA | Intact | NA | NA | NA | NA | NA | Intact | NA | Intact |
| NatA activity | Defect | Defect | NA | Intact | NA | NA | NA | NA | NA | Intact | Defect | Intact |
| NatA thermostability | NA | Defect | NA | NA | NA | NA | NA | NA | NA | NA | Defect | NA |
| NAA10 activity | Intact | NA | Defect | Defect | Defect | NA | NA | Defect | Defect | Defect | NA | Defect |
| NAA10 cellular stability | NA | NA | NA | Defect | NA | NA | NA | Defect | Defect | Defect | NA | Defect |
| Individuals | 2 F | 1 F | 1 F | 1 F | 2 F  1 M | 1 F | 2 F | 6 F | 1 F | 1 F | 1 F | 1 M |
| Number of individuals per variant displaying the following traits |  |  |  |  |  |  |  |  |  |  |  |  |
| Maternal inheritance |  |  |  |  |  |  | NA |  |  |  |  | 1 |
| *De novo* | 2 | 1 | 1 | 1 | 3 | 1 | 1 | 6 | 1 | 1 | 1 |  |
| ID | 2 | 1 | 1 | 1 | 3 |  | 2 | 4 | 1 |  | 1 |  |
| DD | 2 | 1 | 1 | 1 | 3 | 1 | 2 | 6 | 1 | 1 | 1 | 1 |
| Muscular hypotonia |  |  | 1 |  | 3 |  | 1 | 1 | 1 |  |  | 1 |
| Speech delay | 2 |  | 1 | 1 | 3 |  | 2 | 3 |  | 1 | 1 | 1 |
| Feeding difficulties |  | 1 | 1 |  |  |  | 1 | 3 | 1 | 1 | 1 |  |
| Seizures |  |  |  |  | 1 |  |  | 3 |  |  |  |  |
| Brain imaging abnormalities | 1 |  |  |  | 2 |  |  | 4 | 1 | 1 | 1 |  |
| Behavioural abnormalities | 1 | 1 | 1 |  | 3 |  | 1 | 1 |  |  |  | 1 |
| Cardiac anomalies |  |  | 1 |  | 1 |  |  | 1 | 1 |  |  |  |
| Visual impairments | 1 | 1 |  |  |  |  | 1 | 4 | 1 |  | 1 | 1 |
| Microphthalmia |  |  |  |  |  |  |  |  |  |  |  |  |
| Hearing impairments | 2 | 1 |  |  |  |  | 1 |  |  |  |  |  |
| Skeletal abnormalities | 2 | 1 | 1 |  | 2 |  | 1 |  |  |  |  | 1 |
| Genitourinary abnormalities |  |  |  |  | 1 |  |  | 1 |  |  |  |  |
| Growth failure |  |  | 1 |  | 1 |  | 1 | 1 | 1 |  |  |  |
| Short stature | 1 | 1 | 1 |  |  |  |  | 2 |  |  |  |  |
| Microcephaly |  |  | 1 |  |  |  |  | 2 |  | 1 | 1 | 1 |
| Dysmorphic features | 2 |  | 1 |  | 2 |  | 1 |  |  | 1 |  | 1 |

**Table S1 continued.**

| **NAA10 variant** | **c.455_458del** | **c.471+2T>A** | **c.*39A>G** | **c.*40A>G** | **c.*43A>G** | **Total individuals** |
| --- | --- | --- | --- | --- | --- | --- |
| Publication | Shishido *et al.* 2020, Cheng *et al.* 2019 | Forrester *et al*. 2001, Esmailpour *et al.* 2014 | Slavotinek *et al.* 2005, Johnston *et al.* 2019 | Johnston *et al.* 2019 | Graham *et al.* 1991, Johnston *et al.* 2019 |  |
| NatA complex formation | NA | NA | NA | NA | NA |  |
| NatA activity | NA | NA | NA | NA | NA |  |
| NatA thermostability | NA | NA | NA | NA | NA |  |
| NAA10 activity | NA | NA | NA | NA | NA |  |
| NAA10 cellular stability | NA | NA | NA | NA | NA |  |
| Individuals | 2 M | 4 M | 5 M | 1 M | 9 M | **98** |
| Number of individuals per variant displaying the following traits |  |  |  |  |  |  |
| Maternal inheritance | 2 | 4 | 5 | 1 | 9 | **46** |
| *De novo* |  |  |  |  |  | **49** |
| ID | 2 | 4 |  |  | 9 | **66** |
| DD | 1 | 4 |  |  | 9 | **81** |
| Muscular hypotonia | 1 | 4 |  |  |  | **39** |
| Speech delay | 2 | 1 |  |  |  | **53** |
| Feeding difficulties |  |  |  |  |  | **39** |
| Seizures |  | 1 |  |  |  | **22** |
| Brain imaging abnormalities | 1 |  |  | 1 |  | **39** |
| Behavioural abnormalities | 1 |  |  |  |  | **26** |
| Cardiac anomalies | 2 | 4 |  |  |  | **39** |
| Visual impairments |  |  |  |  |  | **33** |
| Microphthalmia | 1 | 4 | 5 | 1 | 9 | **20** |
| Hearing impairments | 1 | 4 | 2 |  |  | **18** |
| Skeletal abnormalities | 1 | 4 | 1 | 1 |  | **40** |
| Genitourinary abnormalities | 2 | 4 |  | 1 |  | **17** |
| Growth failure | 1 |  |  |  |  | **25** |
| Short stature |  |  |  |  |  | **14** |
| Microcephaly |  |  |  |  |  | **17** |
| Dysmorphic features | 1 | 4 | 1 | 1 |  | **48** |

^§^Identified in large size screen
*M, male; F, female; ID, intellectual disability; DD, developmental delay; NA, not available; DDDS, Deciphering Developmental Disorders Study*

**Table S2. Pathogenicity of *NAA10* variants based on ACMG standards**

|  | ***NAA10* p.(A6P)** | ***NAA10* p.(R79C)** | ***NAA10* p.(F128L)** | ***NAA10* p.(Q129P)** | ***NAA10* p.(E157K)** |
| --- | --- | --- | --- | --- | --- |
| **Pathogenicity classification** | **Pathogenic**  *1 Strong (PS1–PS4) AND 2 Moderate (PM1–PM6) AND ≥2 Supporting*  *(PP1–PP5)* | **Pathogenic**  *1 Strong (PS1–PS4) AND 2 Moderate (PM1–PM6) AND ≥2 Supporting*  *(PP1–PP5)* | **Pathogenic**  *≥2 Strong (PS1–PS4)* | **Pathogenic**  *≥2 Strong (PS1–PS4)* | **Likely pathogenic**  *1 Strong (PS1–PS4) AND ≥2 supporting*  *(PP1–PP5)* |
| **Strong evidence** | 1. PS3 Well-established in vitro or in vivo functional studies supportive of a damaging effect on the gene or gene product | 1. PS3 Well-established in vitro or in vivo functional studies supportive of a damaging effect on the gene or gene product | 1. PS1 Same amino acid change as a previously established pathogenic variant regardless of nucleotide change 2. PS2 De novo (both maternity and paternity confirmed) in a patient with the disease and no family history | 1. PS3 Well-established in vitro or in vivo functional studies supportive of a damaging effect on the gene or gene product 2. PS2 De novo (both maternity and paternity confirmed) in a patient with the disease and no family history | 1. PS3 Well-established in vitro or in vivo functional studies supportive of a damaging effect on the gene or gene product |
| **Moderate evidence** | 1. PM1 Located in a mutational hot spot and/or critical and well-established functional domain (e.g., active site of an enzyme) without benign variation 2. PM2 Absent from controls (or at extremely low frequency if recessive) in Exome Sequencing Project, 1000 Genomes Project, or Exome Aggregation Consortium | 1. PM1 Located in a mutational hot spot and/or critical and well-established functional domain (e.g., active site of an enzyme) without benign variation 2. PM2 Absent from controls (or at extremely low frequency if recessive) in Exome Sequencing Project, 1000 Genomes Project, or Exome Aggregation Consortium | 1. PM1 Located in a mutational hot spot and/or critical and well-established functional domain (e.g., active site of an enzyme) without benign variation 2. PM2 Absent from controls (or at extremely low frequency if recessive) in Exome Sequencing Project, 1000 Genomes Project, or Exome Aggregation Consortium | 1. PM1 Located in a mutational hot spot and/or critical and well-established functional domain (e.g., active site of an enzyme) without benign variation 2. PM2 Absent from controls (or at extremely low frequency if recessive) in Exome Sequencing Project, 1000 Genomes Project, or Exome Aggregation Consortium |  |
| **Supporting evidence** | 1. PP2 Missense variant in a gene that has a low rate of benign missense variation and in which missense variants are a common mechanism of disease 2. PP3 Multiple lines of computational evidence support a deleterious effect on the gene or gene product (conservation, evolutionary, splicing impact, etc.) | 1. PP1 Co-segregation with disease in multiple affected family members in a gene definitively known to cause the disease 2. PP2 Missense variant in a gene that has a low rate of benign missense variation and in which missense variants are a common mechanism of disease 3. PP3 Multiple lines of computational evidence support a deleterious effect on the gene or gene product (conservation, evolutionary, splicing impact, etc.) | 1. PP2 Missense variant in a gene that has a low rate of benign missense variation and in which missense variants are a common mechanism of disease 2. PP3 Multiple lines of computational evidence support a deleterious effect on the gene or gene product (conservation, evolutionary, splicing impact, etc.) | 1. PP2 Missense variant in a gene that has a low rate of benign missense variation and in which missense variants are a common mechanism of disease 2. PP3 Multiple lines of computational evidence support a deleterious effect on the gene or gene product (conservation, evolutionary, splicing impact, etc.) | 1. PP2 Missense variant in a gene that has a low rate of benign missense variation and in which missense variants are a common mechanism of disease 2. PP3 Multiple lines of computational evidence support a deleterious effect on the gene or gene product (conservation, evolutionary, splicing impact, etc.) |

**Table S3. NAA10 primers used for mutagenesis**

| **Primer** | **Sequence (5´-3´)** | **Tm (°C)** |
| --- | --- | --- |
| NAA10 c.16G>C p.(A6P) Forward | CATCCGCAATCCGAGGCCAGA | 67 |
| NAA10 c.16G>C p.(A6P) Reverse | TTCATCTAGGAGGCTGAGTC | 63 |
| NAA10 c.235C>T p.(R79C) Forward | GGCTGTGAAGTGTTCCCACCG | 69 |
| NAA10 c.235C>T p.(R79C) Reverse | AATGAGGTGATATGTCCATGGG | 64 |
| NAA10 c.386A>C p.(Q129P) Forward | CTCAACTTTCCGATCAGTGAAG | 55 |
| NAA10 c.386A>C p.(Q129P) Reverse | GGTGTTGGAATAGAGGTG | 59 |
| NAA10 c.469G>A p.(E157K) Forward | GATGGCCGACAAGCTGAGGCG | 70 |
| NAA10 c.469G>A p.(E157K) Reverse | TGAGTGAGGTCCCGCTTC | 66 |

**Table S4. Sequences used for NAA10 multiple sequence alignment**

| Species | UniProt ID | Protein sequence |
| --- | --- | --- |
| *H. sapiens* | P41227 | MNIRNARPEDLMNMQHCNLLCLPENYQMKYYFYHGLSWPQLSYIAEDENGKIVGYVLAKMEEDPDDVPHGHITSLAVKRSHRRLGLAQKLMDQASRAMIENFNAKYVSLHVRKSNRAALHLYSNTLNFQISEVEPKYYADGEDAYAMKRDLTQMADELRRHLELKEKGRHVVLGAIENKVESKGNSPPSSGEACREEKGLAAEDSGGDSKDLSEVSETTESTDVKDSSEASDSAS |
| *M. musculus* | Q9QY36 | MNIRNARPEDLMNMQHCNLLCLPENYQMKYYFYHGLSWPQLSYIAEDENGKIVGYVLAKMEEDPDDVPHGHITSLAVKRSHRRLGLAQKLMDQASRAMIENFNAKYVSLHVRKSNRAALHLYSNTLNFQISEVEPKYYADGEDAYAMKRDLTQMADELRRHLELKEKGKHMVLAALENKAENKGNVLLSSGEACREEKGLAAEDSGGDSKDLSEVSETTESTDVKDSSEASDSAS |
| *R. norvegicus* | D3ZUQ2 | MNIRNARPEDLMNMQHCNLLCLPENYQMKYYFYHGLSWPQLSYIAEDENGKIVGYVLAKMEEDPDDVPHGHITSLAVKRSHRRLGLAQKLMDQASRAMIENFNAKYVSLHVRKSNRAALHLYSNTLNFQISEVEPKYYADGEDAYAMKRDLTQMADELRRHLELKEKGRHMVLSAMENKAENKGNVLLSSGEACREEKGLTAEDSGGDSKDLSEVSETTESTDVKDSSEASDSAS |
| *D. rerio* | Q7T3B8 | MNIRNARPEDLMNMQHCNLLCLPENYQMKYYFYHGLSWPQLSYIAEDENGKIVGYVLAKMEEDPDDVPHGHITSLAVKRSHRRLGLAQKLMDQASRAMIENFNAKYVSLHVRKSNRAALHLYSNTLKFQISEVEPKYYADGEDAYAMKRNLTQMADELQKPGVRLWGSEAPPSQDTSVTGLVEKLTVQDGEKEGDGDSGGESKEMSEVSEATESTDVKDSSSDS |
| *X. laevis* | Q7ZXW3 | MNIRNARPEDLMNMQHCNLLCLPENYQMKYYFYHGLSWPQLSYIAEDENGKIVGYVLAKMEEDPDDVPHGHITSLAVKRSHRRLGLAQKLMDQASRAMIESFNAKYVSLHVRKSNRAALHLYSNTLNFQISEVEPKYYADGEDAYAMKRDLTQMADEQLKKHLEIKEKSRPLSSIENKSDNRSRHVGDCCRDEKCMGNIGKQDLTEDSGDSKDVSEVSEATESTDVKDSSEASDSAS |
| *A. thaliana* | Q9FKI4 | MVCIRRATVDDLLAMQACNLMCLPENYQMKYYLYHILSWPQLLYVAEDYNGRIVGYVLAKMEEESNECHGHITSLAVLRTHRKLGLATKLMTAAQAAMEQVYEAEYVSLHVRRSNRAAFNLYTETLGYKINDVEAKYYADGEDAYDMRKNLKGKQNHHHAHGHHHHHGGGCCSGDAKVVETAQAVDGKAVSK |
| *D. melanogaster* | Q9VT75 | MNIRCAKPEDLMTMQHCNLLCLPENYQMKYYFYHGLTWPQLSYVAVDDKGAIVGYVLAKMEEPEPNEESRHGHITSLAVKRSYRRLGLAQKLMNQASQAMVECFNAQYVSLHVRKSNRAALNLYTNALKFKIIEVEPKYYADGEDAYAMRRDLSEFADEDQAKAAKQSGEEEEKAVHRSGGHGHSHNHSGHDGHCC |
| *S. pombe* | Q9UTI3 | MDIRPARISDLTGMQNCNLHNLPENYQLKYYLYHAISWPMLSYVATDPKGRVVGYVLAKMEEEPKDGIPHGHITSVSVMRSYRHLGLAKRLMVQSQRAMVEVYGAKYMSLHVRKSNRAAIHLYRDTLQFDVQGIESKYYADGEDAYAMHKDFSTLKFDTPETNDELAKTVQSLALNN |
| *S. solfataricus* | Q980R9 | MELAEKDKGRDFTLRNARMDDIDQIIKINRLTLPENYPYYFFVEHLKEYGLAFFVAIVDNSVVGYIMPRIEWGFSNIKQLPSLVRKGHVVSIAVLEEYRRKGIATTLLEASMKSMKNDYNAEEIYLEVRVSNYPAIALYEKLNFKKVKVLKGYYADGEDAYLMARPL |

**
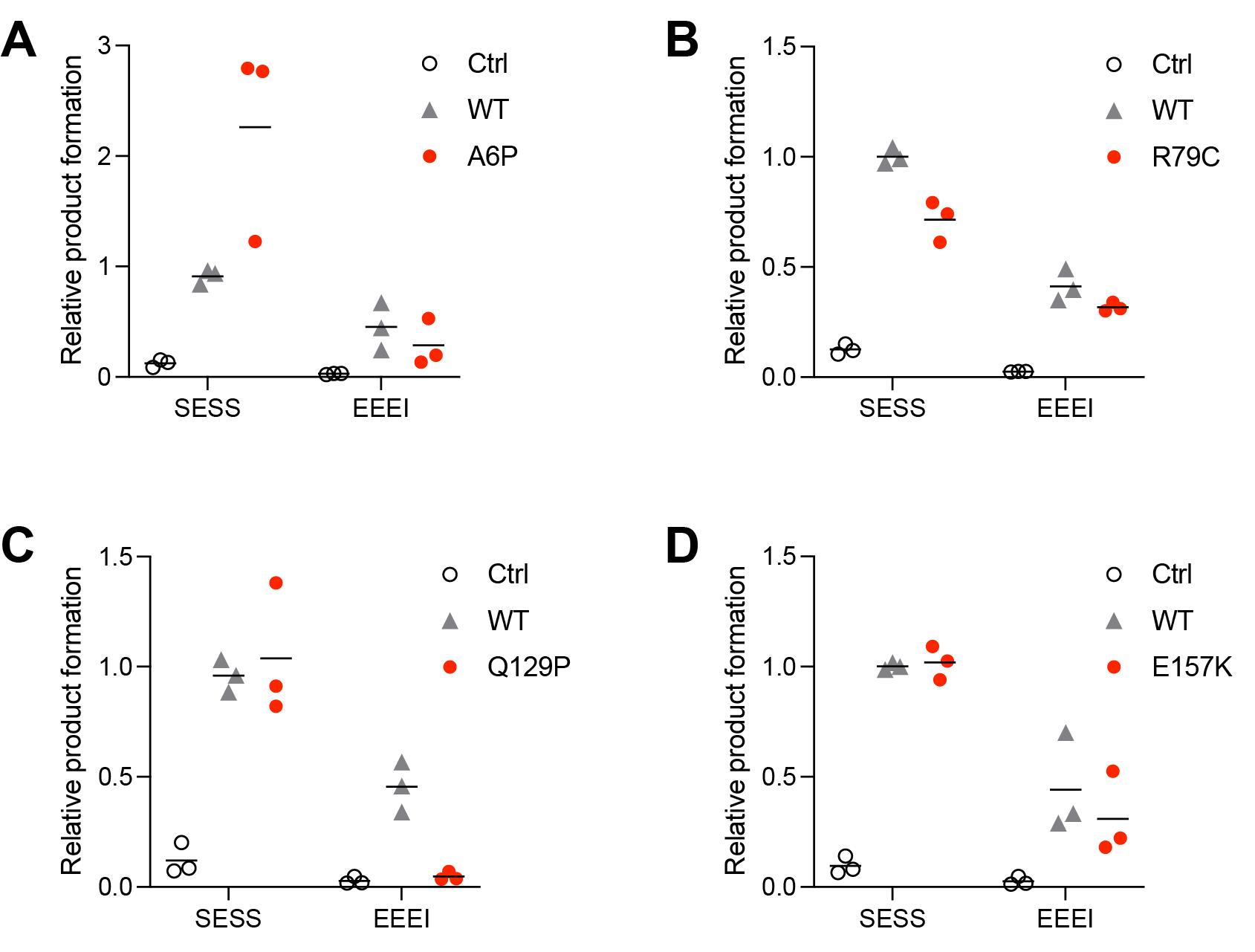
**

**Figure S1. NatA complex formation and N-terminal acetylation by *NAA10* variants.** A-D: Immunoprecipitated NAA10 WT or *NAA10* variants were comparatively tested in Nt-acetylation assays using the NatA substrate SESS and monomeric NAA10 *in vitro* substrate EEEI. β-gal-V5 pull-down was used as input in negative control reactions. The values for Nt-acetylated SESS and EEEI product formation were normalised to Western blot band intensities of NAA15 and NAA10, respectively, and shown as relative to WT. The scatterplots show relative product formation in three independent experiments per variant. The mean of the three independent experiments is indicated by a black line.


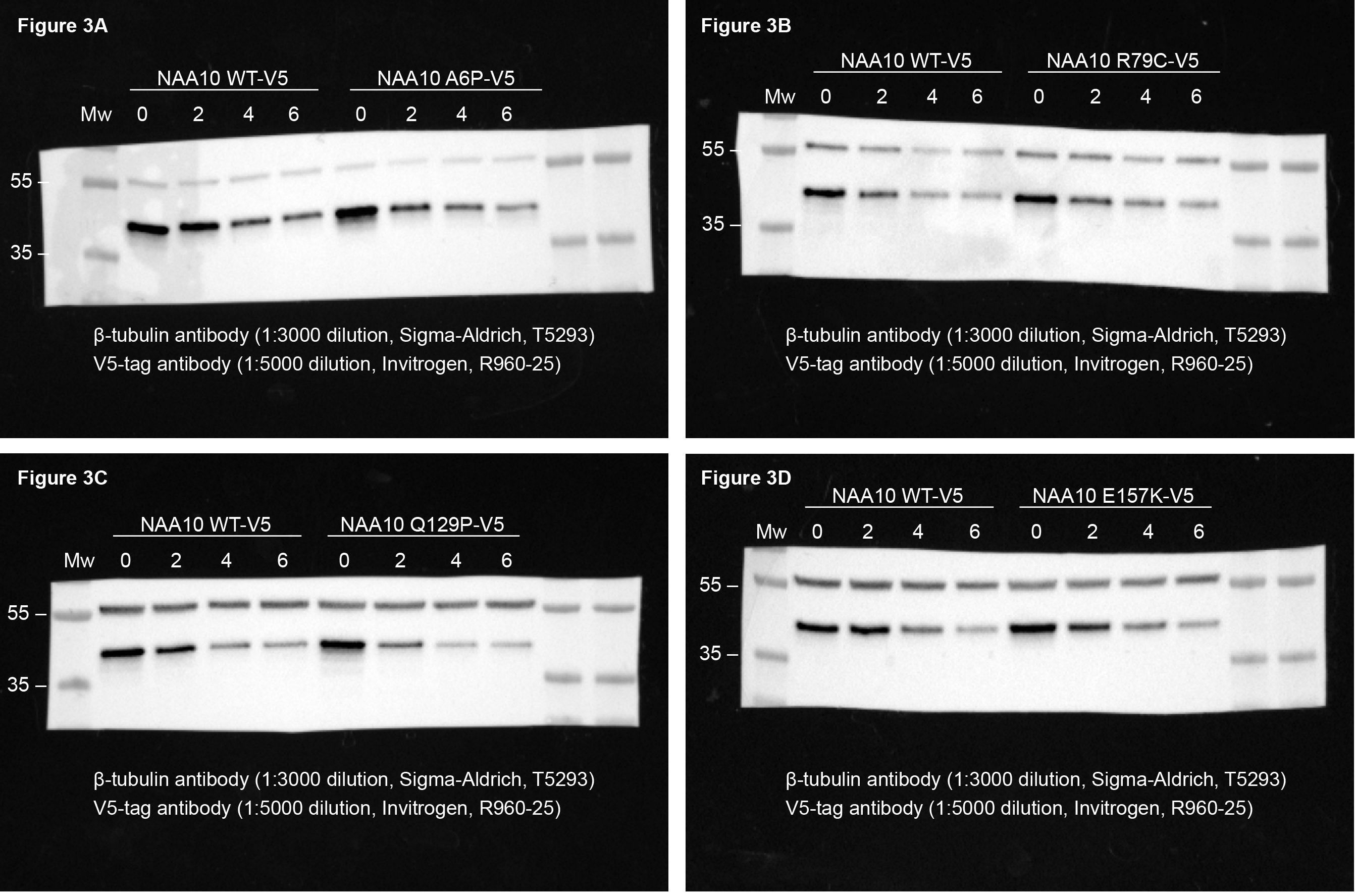


**Figure S2.** Uncropped Western blot images used in main Figure 3.

**
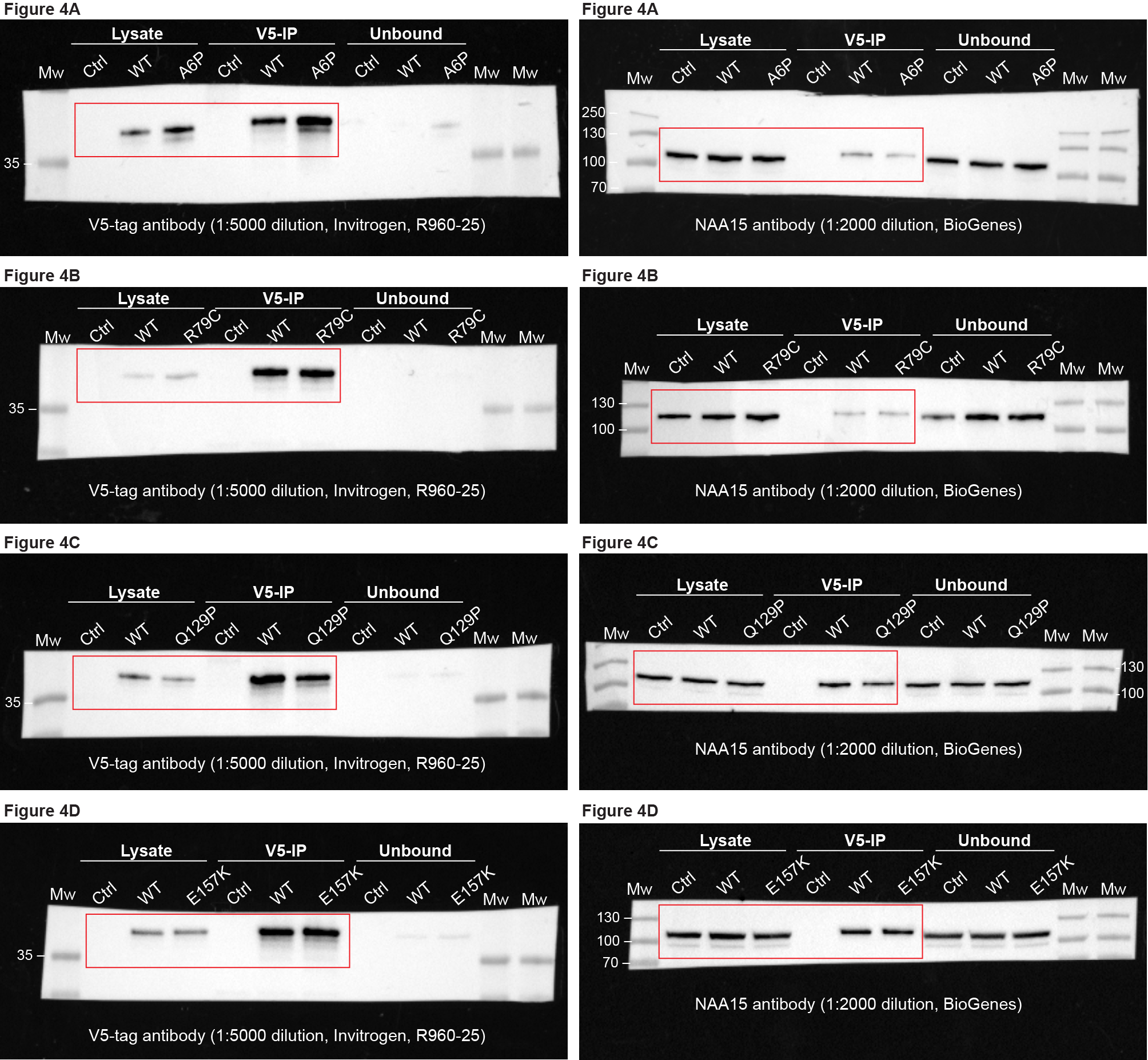
**

**Figure S3.** Uncropped Western blot images used in main Figure 4.
